# Post COVID-19 pulmonary mass: Case report and review of literatures

**DOI:** 10.15537/smj.2022.43.10.20220294

**Published:** 2022-10

**Authors:** Waleed M. Hussen

**Affiliations:** *From the Department of Surgery, College of Medicine, University of Baghdad, Baghdad, Iraq.*

**Keywords:** COVID-19, PCR, bronchoscopy, thoracotomy

## Abstract

To present an unusual and a rare pulmonary affection by coronavirus disease-19 (COVID-19), in which only one lung is affected. Coronavirus disease-19 attacks the lungs and interferes seriously with their functions. The attack is usually bilaterally, while a uni lateral pulmonary affection is unusual. The presentation, both clinical and radiological findings, bronchoscopy appearance, the strange operative findings of the resected mass, the uneventful post-operative course, in addition to the histopathological report, will be presented.

In conclusion, unilateral lung affection is unusual and post-viral pneumonia COVID-19 should be considered as a possible aftermath, which may not be uncommon in Iraq.

**C**oronavirus disease-19 (COVID-19) was considered as pandemic by the World Health Organization (WHO) on March 11, 2020.^1^ The available COVID-19 guidelines deals with the prevention, vaccination, and management. They were based on expert opinions. Recently rapid guidelines on managing the long-term effects of COVID-19 were published to help the clinicians.^2^

Three forms of COVID-19 courses were defined: I) acute (signs and symptoms are present for up to 4 weeks); II) ongoing symptomatic (signs and symptoms are present from 4-12 weeks); and III) post-COVID-19 syndrome (signs and symptoms that develop during and after an infection consistent with COVID-19, are present for >12 weeks and are not attributable to an alternative diagnosis).^2^

The aim of this case report is to preset an unusual and rare presentation of post-COVID-19 pulmonary affection.

## Case Report

A 35-years old male, Iraqi worker, from southern part of Iraq. He presented to the clinic on the 30^th^ of January 2021, with left sided chest pain, that was dull in nature.

### Clinical findings

He was haemodynamically stable namely, normotensive, afebrile. His pulse rate was 82/m of normal sinus rhythm. His chest auscultation was normal. He gave a history of intimate contact with his father, a patient with COVID-19 who had succumbed to the disease. He was treated for COVID-19, as his polymerase chain reaction (PCR) was positive.

### Diagnostic assessment

**Figure 1:**
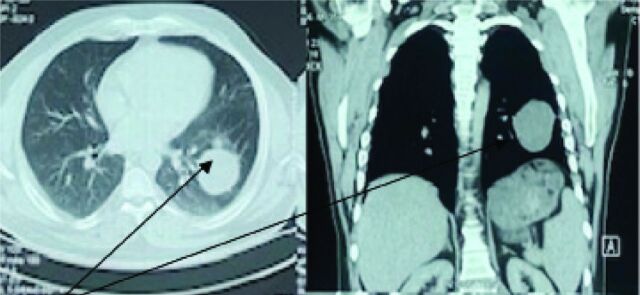
- Computed tomography scan showing a mass in the posterior segment of the left lower lobe.

His chest X-ray showed a clear lesion in the left lower lobe, his earlier chest computed tomography (CT) scan in the beginning of the year 2021 showed a cystic mass with multiple feeders in the left lower lobe. A more recent chest CT demonstrated a dense mass in the left lower lobe with enhancement and no mediastinal lymphadenopathy (Figure 1).

### Therapeutic intervention

Rigid bronchoscopy was carried out under general anesthesia. A bloody carina between the apico-lower and common basal segment was the only finding. Bronchial wash was negative for acid fast bacilli and malignant cells. Biopsy from the area was negative for any evidence of malignancy. The patient was prepared for surgery. Under general anesthesia, double lumen intubation, right lateral decubitus position, and classical left thoracotomy approach, the chest was entered through 5^th^ intercostal space.

**Figure 2:**
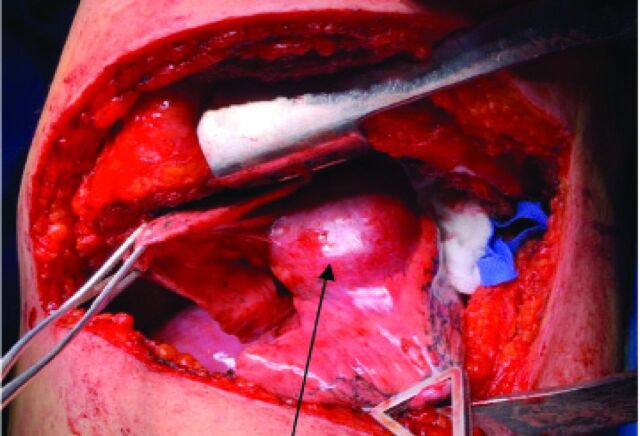
- An operative view of the mass.

**Figure 3:**
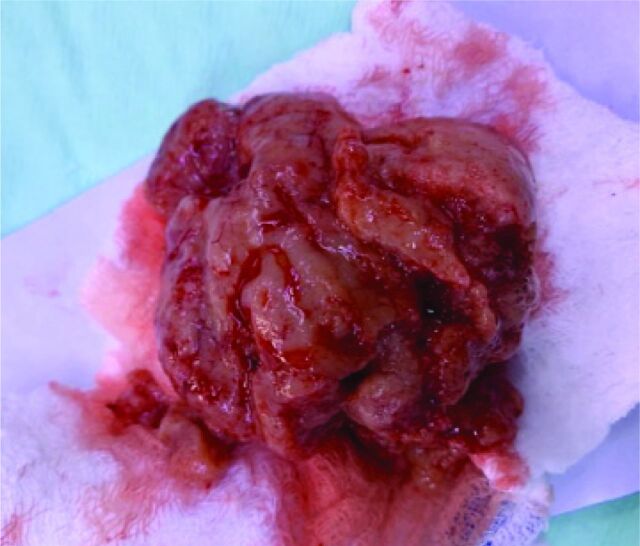
- The contents of the mass.

A mass was visible involving the apico-lower segment and projecting into the fissure between the left upper lobe and left lower lobe (Figure 2). The mass was removed completely. Its content was a fleshy gelatinous material (Figure 3). The chest was closed in the standard way, leaving a single chest tube.

### Follow-up and outcome

The post-operative course was uneventful, and the chest X-ray showed a cleared left lung.

The histopathological report stated an interstitial and intra-alveolar proliferation of fibroblasts, pneumocytes, some were multinucleated, with lymphocytic infiltration forming lymphoid aggregate, the picture is compatible with post-viral pneumonitis (possibly post COVID-19) and not with malignancy.

Patient attainted a regular follow-up clinic, with complete disappearance of symptoms and with normal chest X-ray. Patient’s summarized time line is shown in Table 1.

## Discussion

An infected person with COVID-19 virus can transmit the virus within 24-48 hours prior to the appearance of symptoms.^3^ The patient was an intimate contact to his father who succumbed to COVID-19. He received treatment for COVID-19 as his PCR was positive, but no imaging study was carried out for him and this might be explained by the unpreparedness of the health system in Iraq for the pandemic in early 2020.

Chest CT was carried out early in 2021, on the last consultation, a dense mass was found. The lack of accumulated experience on the manifestations of COVID-19 in the chest CT scan might be an explanation for not pointing to the possibility of COVID-19. Nowadays, many articles have appraised the radiological pattern of different forms of diffuse parenchymal lung disease during different courses of COVID-19.^4,5^ Coronavirus disease-19 pneumonia manifested on chest CT scan imaging abnormalities, even in asymptomatic patient. It rapidly evolutes from focal unilateral and progresses to or coexists with consolidation within 1-3 weeks.

**Table 1:** - Patient’s summarized timeline.

Dates	Relevant past medical history and interventions	
	**Diagnostic testing**	**Therapeutic intervention**
July 2020	PCR for COVID-19 was positive	Symptomatic and supportive treatment
January 2021	Imaging testing	Symptomatic treatment
February 2021	Re CT chest	Rigid bronchoscopy under GA
April 2021	Pre-operative basic investigation and blood preparation	Left thoracotomy and removal of the left lower lobe mass
September 2021	Chest X-ray	No treatment needed

PCR: polymerase chain reaction, COVID-19: coronavirus disease-19, CT: computed tomography, GA: general anesthesia

The reports of COVID-19 related lung pathology among asymptomatic individuals at the time of surgery is rare in the literature.^6^ This case report throws a light on the lung pathology among asymptomatic COVID-19 patient. A similar event was reported as a COVID-19 case was diagnosed after right lower lobectomy for removal of a pulmonary nodule.^7^ Emendation on CT scan findings of COVID-19 pneumonia was published in Egypt.^8^ An assay on the radiological findings of possible or confirmed COVID-19 was published in Turkey.^9^

The limited experience in video assisted thoracoscopic surgery was the reason behind utilizing the standard thoracotomy approach for removal of the mass.

The histopathological picture was that of post-viral pneumonitis. The literature documented that clinicians have not sufficiently considered the condition of viral induced secondary organizing pneumonia which in turn appears in the publications on COVID-19.^10^ This case report contributes to the knowledge regarding COVID-19.

In conclusion, unilateral lung affection is unusual and post-viral pneumonia COVID-19 should be considered as a possible aftermath, which may not be uncommon in Iraq.
